# The expression of the tight junction protein and therapeutical target Claudin 18.2 is heterogeneously distributed within esophageal and gastric adenocarcinoma

**DOI:** 10.1038/s41598-025-12337-4

**Published:** 2025-08-07

**Authors:** Svetlana Pak, Adrian Georg Simon, Su Ir Lyu, Yuri Tolkach, Hakan Alakus, Thomas Zander, Reinhard Buettner, Christiane Josephine Bruns, Wolfgang Schroeder, Alexander Quaas

**Affiliations:** 1 Institute of Pathology, Medical Faculty, University Hospital Cologne, University of Cologne, Kerpener Str. 62, 50937 Cologne, Germany; 2 Department of Internal Medicine I, Medical Faculty, University Hospital Cologne, University of Cologne, Cologne, Germany; 3 Department of General, Visceral and Cancer Surgery, Medical Faculty, University Hospital Cologne, University of Cologne, Cologne, Germany

**Keywords:** Esophageal cancer, Zolbetuximab, Claudin, Tight junctions, Gastric cancer, Biomarkers, Oncology

## Abstract

Claudin 18.2 (CLDN18.2) is a therapeutically relevant biomarker in esophageal (EAC) -and gastric adenocarcinoma (GAC). Little is known about its heterogeneity within the primary tumor and corresponding metastases and how many biopsies are needed to determine the true CLDN18.2 status of a tumor. CLDN18.2 was assessed in 1,283 patients (822 EAC, 461 GAC), using the antibody clone 43–14 A. Eight virtual endoscopic biopsies were taken from digitized whole tumor blocks. 204 of 822 EAC (24.8%) and 132 of 461 GAC (28,6%) were positive for CLDN18.2. In GAC, CLDN18.2 expression showed a trend towards less invasive growth (*p* = 0.02) and less common lymph node metastasis (*p* = 0.07) and was more often observed within the EBV-associated subtype (*p* = 0.01). When comparing the expression in one biopsy with the whole tissue section, the sensitivity for CLDN18.2 was low (58.8%). The sensitivity increased to a maximum of 76.5% with 6 and 8 biopsies (positive likelihood ratio = 17.8). Discordant expression between primary tumor and corresponding local lymph node metastasis was observed in 18.5%. This study highlights that CLDN18.2 is a heterogeneously expressed biomarker, within the tumor tissue as well as in primary tumor and corresponding lymph node metastasis. In case of CLN18.2-negative results the representativity of the biopsies must be critically assessed and a re-biopsy might be recommendable.

**Supplementary Information:**

The online version contains supplementary material available at 10.1038/s41598-025-12337-4.

## Introduction

Esophageal adenocarcinoma (EAC) and gastric adenocarcinoma (GAC) are among the most aggressive malignant epithelial tumors, taking the 7th place in mortality in male patients while being relatively uncommon in women^1^. The typical treatment of patients with resectable, non-metastatic disease consists of chemotherapy, usually combined with radiotherapy, followed by surgical resection^2^. In 2021, the CheckMate 577 study demonstrated that adjuvant treatment with nivolumab improved the disease-free survival in patients with residual cancer, independent of the status of tissue biomarkers^3^. In a primary palliative setting, the expression of PD-L1- and Her2neu in tumor tissues might trigger targeted therapy with immune-checkpoint inhibitors or trastuzumab, respectively, however, a platinum-based chemotherapy remains the therapeutical standard^2^. When in advanced or metastasized stage, the prognosis is dismal, patients usually survive few months^4,5^. Thus, there is an urgent need for targeted therapies and new therapeutic approaches, especially in the palliative setting.

The tight junction protein Claudin 18.2 (CLDN18.2) was shown to be a highly selective biomarker aberrantly overexpressed in oesophageal adenocarcinoma with limited expression in benign mucosal tissue^6^. With this specific expression pattern, CLDN18.2 is a feasible target for the development of therapeutic substances. The investigated CLDN18.2-targeting therapeutic agents so far include monoclonal antibodies (Ab), bispecific Ab, chimeric antigen receptor T (CAR-T) cells, and Ab-drug-conjugates (ADC)^6,7^.

In November 2022, Zolbetuximab was granted a fast-track status by Food and Drug association (FDA) and was also filed by European Medical Agency (EMA) as a first-in-class therapeutic monoclonal antibody against CLN18.2 for therapy of HER2/neu-negative upper gastrointestinal tract carcinomas based on the submitted phase 3 clinical study results^8^. In course of this trial, immunohistochemical staining was established as a predictive biomarker, with expression of CLND18.2 expressed in more than 75% of tumor cells being a pre-requisite for therapy. Although CLDN18.2 was shown to be a predictive marker in this clinical study, little is known about its expression patterns in larger, real-world patient populations, with especially esophageal adenocarcinoma being known for its heterogeneity in geographical distribution, clinical course and biology at the tissue and cell level^2^.

The aim of this study is to investigate the tissue expression of CLDN18.2 in a large, mainly Caucasian cohort of EAC- and GAC patients, with a focus on the intratumoral heterogeneity of CLDN18.2 expression, and its potential impact on the indication for a targeted therapy in the biopsy setting is assessed.

## Materials and methods

### Patient cohort of EAC and GAC

**Fig. 1:**
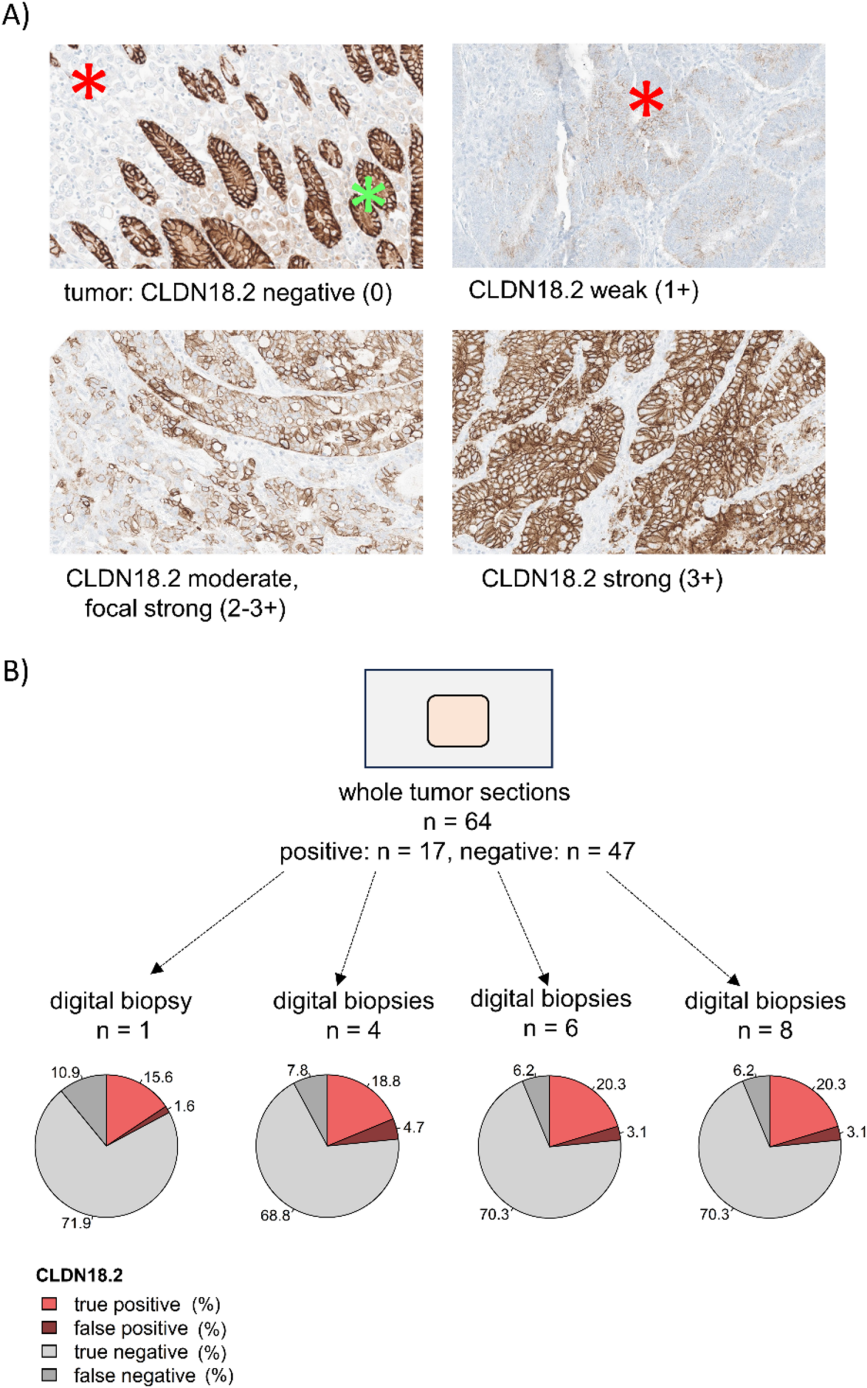
CLDN18.2 expression in whole tissue sections and digital biopsies. (**A**) Immunohistochemical expression differences of claudin 18.2 (Ventana-clone 43–14 A). A tumor was considered positive, if at least 75% of tumor cells had a moderate (2+) or strong (3+) CLDN18.2 expression; red asterix: tumor tissue, green asterix: benign esophageal glands (internal positive control); magnification 200x. (**B**) 17 whole-tumor sections were positive for CLDN18.2 (26.6%). When one single digital biopsy was randomly taken from the tumor, 10/17 tumors were correctly identified as positive (sensitivity 58.8%); The sensitivity increased to 76.5%, when 6 or 8 digital biopsies were taken, respectively (Table 2).

For immunohistochemical analysis of CLDN18.2 expression, tumor tissue from 822 with operable EAC and 461 cases of operable GAC were obtained (n total = 1283). Follow-up data were available (Fig. 1; Table 1).

For EAC, patients were surgically treated with right transthoracic esophagectomy and a two-field lymphadenectomy (mediastinal and abdominal lymph nodes). The intestinal passage was reconstructed with a high intrathoracic esophagogastrostomy as described previously^9^.

Patients with GAC were treated with a subtotal distal or total gastrectomy with trans-hiatal resection of the distal esophagus in case of a Siewert II-tumor, followed by lymphadenectomy (level D2). A Roux-en-Y jejunal loop with gastrojejunostomy was the method for intestinal reconstruction. In EAC, neoadjuvant therapy consisted of either radio-chemotherapy according to the Chemo-radiotherapy for Oesophageal Cancer followed by Surgery Study (CROSS) protocol (carboplatin, paclitaxel and intensity-modulated radiotherapy) or perioperative chemotherapy according to FLOT (Fluorouracil, Leucovorin, Oxaliplatin and Docetaxel). For the treatment of patients with GAC, three different perioperative regimens were used in the last two decades: Cisplatin, 5-Fluorouracil and Leucovorin (PFL), Medical Research Council Adjuvant Gastric Infusional Chemotherapy according to MAGIC (Epirubicin, Cisplatin, Fluorouracil) and FLOT. Most patients were treated with MAGIC and FLOT protocols according to national German guidelines.

In the first two years after surgery, a clinical follow-up was performed every three months, followed by annual check-ups. The clinical follow-up included a detailed update and record of the patients’ medical history, physical examination, an ultrasound scan of the abdomen, a chest X-ray, and if required, additional diagnostics in individual cases.

The study protocol was in accordance with the ethical guidelines of the 1964 Declaration of Helsinki and its later amendments as reflected by the approval of the institution´s review committee (Ethic Committee of the Medical Faculty of University of Cologne: registration no. 13–091; ethic committee no. 21-1146). All patients gave written informed consent to the use of their tumour specimen and their data for research and publication.

### Tissue microarray generation and whole tissue sections

To assess the expression and intratumoral distribution of CLDN18.2 in EAC and GAC samples, various tissue microarrays (TMAs) were generated as reported previously^10,11^: Shortly summarized, one tissue core with a diameter of 1.2 mm was transferred to the recipient paraffin block with a self-constructed, semi-automated precision instrument. Placenta tissue was included as a control.

We generated single spot TMAs with one core of each patient (*n* = 1,283): These TMAs consisted of primary tumors as well as corresponding lymph node metastases. For 763 patients, only tumor tissue was available; For 520 patients, primary tumor tissue and a biopsy of the correspondent lymph node metastasis were available.

Additionally, multi-spot TMAs with several cores from superficial and deep tumor parts of 165 patients were generated: In these TMAs, the primary tumor is represented by 8 tumor spots: 4 tumor spots were taken from endoscopically accessible, superficial tumor areas, 4 additional cores were taken from more central parts and the deep infiltration zone of the tumor. Regional lymph node metastases were included with 4 additional cores, allowing a full-scale assessment of the heterogeneity of the CLDN18.2 expression.

In cases with discrepant CLDN18.2 expression between primary tumors and lymph node metastasis in the tissue microarrays, we re-assessed whole-tumor sections of 25 EAC and 25 GAC.

### Immunohistochemical assessment of CLDN18.2 expression as well as PD-L1 and Her2/neu expression

To assess the CLDN18.2 expression we used the antibody-clone of the Spotlight-study (clone: 43–14 A-assay, ready-to use, mouse; Ventana/Roche, Switzerland) on a Ventana Benchmark staining platform. The assessment of the CLDN18.2 expression followed the evaluation criteria of the Spotlight study: The extent of CLDN18.2 expression was determined using a semi-quantitative approach. Only membranous staining (complete and incomplete) of tumor cells was considered. A tumor was considered CLDN18.2-positive if at least 75% of the available tumor cells expressed CLDN18.2 at a moderate or strong staining intensity (Fig. 1). Normal gastric mucosa was used as a positive control as recommended by Ventana. The mucosa always showed a strong (score 3) membranous CLDN18.2 positivity.

The Her2/neu status (clone: 4B5, Ventana/Roche, ready to use on Ventana Benchmark) and PD-L1-CPS-status (clone: E1L3N; Cellsignaling (Danvers, USA); 1:400 EDTA on Leica Bond (Wetzlar, Germany)). Her2/neu status as described previously^12,13^. Since the treatment of zolbetuximab is only approved for the treatment of Her2/neu negative tumors, we performed the Claudin 18.2 status as well as PD-L1 status on the whole tumor blocks only on Her2/neu negative tumors.

In cases of GAC, the molecular subtypes according to the TCGA classification (chromosomal instable (CIN), genomic stable (GS), microsatellite-instable (MSI) and EBV-associated (EBV) were determined as described before^10^.

### Digital generation of 8 endoscopy-like biopsies from whole-tumor sections

For generation of digital, endoscopy-like biopsies, whole-tumor sections from *n* = 64 primary operated tumors (40 gastric adenocarcinoma and 24 esophageal adenocarcinoma) were digitized and assessed for the CLDN18.2 expression. The tumors analyzed here were Her2/neu negative (Dako score 0 or score 1). All histological slides were digitized by Hamamatsu Nanozoomer S360 histoscanner (Hamamatsu Photonics, Hamamatsu, Japan) at 400x magnification.

The CLDN18.2 expression obtained on the whole-tumor sections served as the gold standard (= ground truth), with the whole tumor being assessable.

From these sections, 8 digital biopsies were punched out from endoscopically accessible, superficial tumor areas with a specific algorithm: For this purpose, the superficial parts, which are accessible for endoscopic biopsies (the tumor surface down to upper third of the submucosa layer) were annotated manually by an experienced human analyst using the QuPath software (QuPath v. 0.3.2). Further, a computational algorithm was created in Python 3.9 that performed the random virtual biopsies in a random manner: A regularization parameter prevented taking all biopsies from one location within tumor, which can occur during fully random sampling. This parameter functioned as follows: The ideal distance between a selected number of biopsies was calculated for a defined number of biopsies, which ideally cover the whole tumor with the same distances between single biopsies; The distance between each random biopsy then was determined to be ≥ 50% of this ideal distance. This also mirrors the endoscopic practice, as endoscopists will attempt to cover the whole tumor with biopsies. Additionally, the digital biopsy size corresponded to the real biopsy size (1.8 mm) as obtained by the endoscopes (GIF-H 190, Olympus Tokyo Japan) of our department of gastroenterological endoscopy.

The digital, virtual biopsies were further digitally assessed by an experienced pathologist for the CLDN18.2 expression.

### Statistical analysis and survival analysis

All data processing and statistical analysis, including survival analysis and visualization, were performed with R (v.4.2.2) and Rstudio (v. 2022.12.0 + 353) with common free packages including survival (v.3.4-0), survminer (v.0.4.9), and ggplot2 (v.3.4.0). Interdependencies between clinical data, histopathological data and CLDN18.2 expression were evaluated using Fisher’s exact test and Spearman’s correlation test with a Bonferroni correction for multiple comparisons. To assess the correlation between CLDN18.2 expression in EAC and GAC and its association with the outcome, overall survival (OS) was evaluated from the date of surgery until death (of any cause). Kaplan Meier curves were generated and a log-rank test was used. Patient data with no events or patients lost to clinical follow-up were censored at the last known date. For multivariate analysis, covariates were implemented using the ENTER method in a Cox proportional hazard model. Covariates which were significant in univariate analysis were included in the multivariate model. A p-value ≤ 0.05 was considered to be significant in all tests.

**Table 1:** Patient cohorts of esophageal and gastric adenocarcinomas.

	EAC (*n* = 822)	GAC (*n* = 461)
	*n* (%)	*n* (%)
Sex		
Male	720 (87.6)	308 (66.8)
Female	102 (12.4)	153 (33.2)
Median age (range)		
pT		
pTx	12 (1.5)	1 (0.2)
pT1	156 (19.0)	57 (12.4)
pT2	144 (17.5)	148 (32.1)
pT3	482 (58.6)	181 (39.3)
pT4	28 (3.4)	74 (16.1)
pN		
pNx	12 (1.5)	2 (0.4)
pN0	326 (39.7)	161 (34.9)
pN1+	484 (58.9)	298 (64.4)
L		
Lx	156 (19.0)	68 (14.8)
L0	364 (44.3)	147 (31.9)
L1	302 (36.7)	246 (53.4)
V		
Vx	151 (18.4)	68 (14.8)
V0	593 (72.1)	338 (73.3)
V1	78 (9.5)	55 (11.9)
Pn		
Pnx	147 (17.8)	68 (14.8)
Pn0	511 (62.2)	297 (64.4)
Pn1	164 (20.0)	96 (20.8)
M		
Mx/M0	816 (99.3)	386 (83.7)
M1	6 (0.7)	75 (16.3)
Neoadjuvant treatment*		
NA	12 (1.5)	
Yes	522 (63.5)	319 (69.2)
No	288 (35.0)	142 (30.8)
AJCC Grade**		
NA	538 (65.5)	142 (30.8)
1	2 (0.2)	3
2	147 (17.9)	114
3	131 (15.9)	193
4	4 (0.5)	9
TCGA molecular subtype ***		
NA	–	52 (11.3)
EBV	–	24 (5.2)
MSI-high	–	37 (8.0)
GS	–	43 (9.3)
CIN	–	305 (66.2)
CLDN18.2 expression		
Negative	618 (75.2)	329 (71.4)
Positive	204 (24.8)	132 (28.6)

*For EAC, neoadjuvant therapy consisted of either radiochemotherapy according to the Chemo-radiotherapy for Oesophageal Cancer followed by Surgery Study (CROSS) protocol (carboplatin, paclitaxel and intensity-modulated radiotherapy) or perioperative chemotherapy (Fluorouracil, Leucovorin, Oxaliplatin and Docetaxel: FLOT). Patients with GAC received different perioperative regimens in the last two decades: Cisplatin, 5-Fluorouracil and Leucovorin (PFL), Medical Research Council Adjuvant Gastric Infusional Chemotherapy (Epirubicin, Cisplatin, Fluorouracil: MAGIC) and FLOT. Most patients were treated with MAGIC and FLOT protocols according to national German guidelines; ** AJCC Grading was only performed for tumors without neoadjuvant therapy; Molecular subtype, according to the molecular TCGA subtype classification of gastric adenocarcinoma (Cancer Genome Atlas Research 2014): EAC = esophageal adenocarcinoma, GAC = gastric adenocarcinoma; median age = median age at diagnosis; pT = tumor stage, pN = lymph node stage, L = lymph vessel invasion, V = blood vessel invasion, Pn = perineural invasion; M stage = distant metastasis; NA = not analyzed, EBV = EBV-associated GAC, MSI-high = microsatellite-instable subtype, GS = genomic stable subtype, CIN = chromosome instability subtype.

## Results

### CLDN18.2 expression in esophageal and gastric adenocarcinomas

In the total cohort, 204 of 822 esophageal adenocarcinomas (EAC) were positive for CLDN18.2 (24.8%).

In patients treated with primary surgery (*n* = 288), 75 EAC expressed CLDN18.2 (26.0%). In the cohort after neoadjuvant (radio)chemotherapy (*n* = 522), 129 tumors were positive (24.7%). The expression of CLDN18.2 was not associated with patients’ age or sex. No correlation was observed between tumor stage (pT), lymph node status (pN), lymphatic invasion (L), blood vessel invasion (V), perineural invasion (Pn), the presence or absence of distant metastasis (M) (all *p* > 0.05).

In the total cohort of gastric adenocarcinoma (GAC),132 of 461 tumors were positive for CLDN18.2 (28.6%). These proportions were also observed for treatment-naïve tumors (positive: 93/319, 29.2%) and tumors treated with perioperative therapy (positive: 39/142, 27.5%). CLDN18.2-positive gastric adenocarcinomas were significantly less often invasive beyond the mucosa (pT2 and higher; Fisher’s exact test, *p* = 0.008). Additionally, patients with CLDN18.2-positive tumors less often had lymph node metastasis (pN+; Fisher’s exact test, *p* = 0.02). After Bonferroni correction for multiple comparisons, CLDN18.2-positive tumors still trended towards less invasive growth (pT2+, *p* = 0.06) and less lymph node metastasis (*p* = 0.07).

No difference in overall survival was observed between CLDN18.2-positive and CLDN18.2-negative EAC- and GAC patients, neither in the total cohorts nor in the treatment-naïve cohorts or the cohorts with neoadjuvant/perioperative treatment (Supplementary Fig. 1).

### Expression of CLDN18.2 is more common in EBV-associated gastric cancers

When the distribution among the molecular subtypes and CLDN18.2 expression was analyzed, CLDN18.2 positive tumors more often belonged to the EBV-associated subtype of GAC than CLDN18.2-negative tumors with 14/24 tumors (58.0%) expressing CLDN18.2 (*p* = 0.01). In the genomic stable (GS) subgroup, which includes in particular the poorly cohesive and signet ring cell differentiated adenocarcinomas 11/49 GAC (22.4%) expressed CLDN18.2, in the chromosomally instable (CIN) subgroup 91/337 GAC (27.0%) and in the MSI subgroup 12/40 GAC (30%), respectively.

Correlation of CLDN18.2 with PD-L1 CPS status.

The PD-L1 CPS score was also determined on the whole tumor blocks (*n* = 66). In the group of CLDN18.2 positive tumors, 8 tumors also showed PD-L1 positivity with an expression of CPS 1 or higher (44%) (PD-L1 CPS 1–4 = 3, CPS 5–10 = 5, CPS > 10 = 0). If a PD-L1 expression level of CPS 5 is used (as relevant for approval in the Checkmate 649 study for nivolumab), then 5 tumors show a corresponding PD-L1 expression level (27%) (supplement Fig. 2).

### Single spot biopsies have a low sensitivity for the assessment of CLDN18.2 expression

For 64 adenocarcinoma (40 gastric adenocarcinoma and 24 esophageal adenocarcinoma), whole-tumor sections were stained for CLDN18.2.

17/64 tumors were CLDN18.2-positive in the whole-tumor section cohort (26.6%).

However, when comparing the results of only one single core biopsy used in single-spot TMA with the whole-tumor sections, the sensitivity for CLDN18.2 expression was low (58.8%). The specificity was 97.9%.

In the next step, we re-assessed the sensitivity and specificity, when 8 endoscopy-like digital biopsies were taken from the whole-tumor slide: If ≥ 75% of the cores were positive (e.g., 3 out of 4 cores, 5 out of 6 cores, 6 out of 8 cores), the tumor was considered positive. The sensitivity increased to a maximum of 76.5% with 6 or 8 biopsies, while the specificity slightly declined to 95.7%. The proportion of false-negative classified tumors (the tumor was classified as CLDN18.2-positive when analyzing the whole-tumor sections but was misclassified as negative when analyzing individual biopsies from the tumor) was 10.9% when considering one single biopsy. This value decreased to 6.2% when 6 or 8 biopsies were available for analysis. Conversely, the proportion of tumors rated falsely positive increased from 1.6% (*n* = 1 tumor) when considering the single biopsy to 3.1% (*n* = 2 tumors) when considering six or eight biopsies, respectively (Fig. 1; Table 2). Both for 6 and 8 biopsies, the positive likelihood ratio (LR+) was 17.8, while the negative likelihood ratio was 0.25 (Table 2).

**Table 2:** Sensitivity and specificity of digital core biopsies for CLDN18.2 expression.

	1 biopsy		4 biopsies		6 biopsies		8 biopsies	
	Pos.	Neg.	Pos.	Neg.	Pos.	Neg.	Pos.	Neg.
Tumor: CLDN18.2 positive (*n* = 17 (26.6%))	10	7	12	5	13	4	13	4
Tumor: CLDN18.2 negative (*n* = 47 (73.4%))	1	46	3	44	2	45	2	45
Sensitivity	58.8% (10/17)		70.6% (12/17)		76.5% (13/17)		76.5% (13/17)	
Specificity	97.9% (46/47)		93.6% (44/47)		95.7% (45/47)		95.7% (45/47)	
LR+	28.0		11.0		17.8		17.8	
LR-	0.4		0.3		0.25		0.25	

pos. = CLDN18.2 positive, neg. = CLDN18.2 negative; LR + positive likelihood ratio, LR- negative likelihood ratio.

### Biopsies of superficial and central tumor regions reveal a relevant heterogeneity in CLDN18.2 expression

**Fig. 2:**
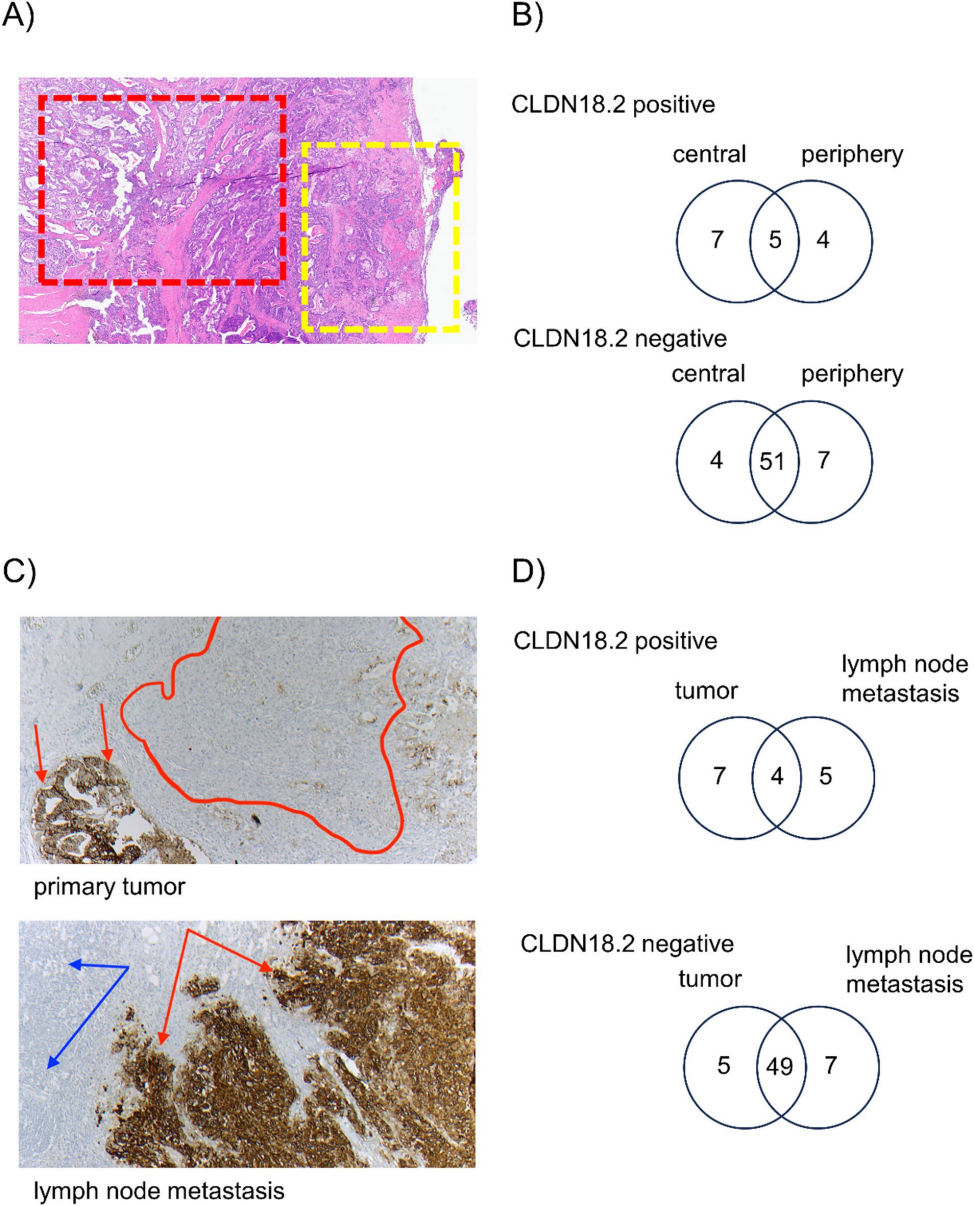
Expression of CLDN18.2 in central tumor, peripheral tumor and lymph node metastases. (**A**) Overview from a whole tumor slide: four tissue punches for the heterogeneity array were biopsied (diameter 1.2 mm) from peripheral tumor regions close to the surface (yellow), which could also be reached by conventional endoscopic biopsies. Another four TMA punches were taken from the depth of the tumor (red), near the deepest invasion site of the tumor. This area cannot be reached by endoscopic biopsies. In addition, four TMA punches were obtained from available lymph node metastases (not shown here). magnification 30x. (**B**) For positive tissue samples, a poor concordance was observed, with more tumors being positive when biopsies were taken from the central/deep tumor areas (12 vs. 9 tumors). For negative tumor samples, a higher concordance was observable. Thus, 56/67 tumors (83.6%) displayed a concordant CLDN18.2 expression (CLDN18.2 positive or negative). (**C**) Intra- and intertumoral heterogeneity of CLDN18.2. In the tumor (upper picture) there are areas which show a strong CLDN18.2 expression (3+) (long red arrows) and larger sections that are completely negative (circled in red). In between are small tumor cell clusters with weak CLDN18.2 expression (1+). Overall, this tumor showed < 75% CLDN18.2 positive tumor cells (CLDN18.2 negative). The corresponding lymph node metastasis (bottom picture) shows a strong, diffuse CLDN18.2 expression (3+) in 100% of the tumor cells (red arrows); Thus, the lymph node metastasis is CLDN18.2 positive. Lymphatic tissue of the lymph node shown with blue arrows; magnification 50x. (**D**) For 65 tumors, matched lymph node metastases were available. 4 biopsies from the tumor (central cores and/or superficial) were compared to 4 biopsies taken from the lymph node metastases. In 53/65 cases (81.5%), primary tumor and lymph node metastasis displayed a concordant CLDN18.2 expression (CLDN18.2 positive or negative).

In the next step we evaluated the CLDN18.2 expression in 4 core biopsies taken from the superficial/central parts of the tumor and compared them with 4 biopsies taken from the central tumor areas/deep infiltration area in 67 tumors (Fig. 2).

A total concordance in CLDN18.2 expression was observed in 56 tumors (83.6%), 11 tumors displayed a discrepancy of CLDN18.2 expression between superficial and deep tumor areas (16.4%).

Central/deep biopsies were more often positive for CLDN18.2 than superficial biopsies (12/67 (17.9%) vs. 9/67 (13.4%) (Table 3).

**Table 3:** CLDN18.2 expression in superficial and central biopsies.

		4 peripheral biopsies			
		CLDN18.2 positive	CLDN18.2 negative	n total	
4 central biopsies	CLDN18.2 positive	**5**	7	12	Positive agreement rate: 41.7%
	CLDN18.2 negative	4	**51**	55	Negative agreement rate: 92.7%
	n total	9	58	67	Overall agreement rate: 83.6%

### Primary tumors and corresponding lymph node metastases display a relevant heterogeneity of CLDN18.2 expression

We additionally compared 4 biopsies from tumor tissue with 4 biopsies taken from the corresponding lymph node metastasis. 9/65 tumor samples (13.8%) and 11/65 lymph node metastases (16.9%) were positive for CLDN18.2. When both samples were compared, the lymph node metastasis displayed the CLDN18.2 expression of the tumor tissue of origin in 53/65 cases (81.5%). In 12 patients, a discordant expression was observed (18.5%) (Table 4).

**Table 4:** CLDN18.2 expression in tumor biopsies and corresponding lymph node biopsies.

		*n* = 4 lymph node biopsies			
		CLDN18.2 positive	CLDN18.2 negative	n total	
*n* = 4 Tumor biopsies	CLDN18.2 positive	**4**	7	11	Positive agreement rate: 36.4%
	CLDN18.2 negative	5	**49**	54	Negative agreement rate: 90.7%
	n total	9	56	65	Overall agreement rate: 81.5%

## Discussion

In this study, we analyzed almost 1300 adenocarcinomas of the upper GI tract in a predominantly Caucasian patient population. The patients were treated in one of the largest tumor centers in Germany, specialized in the therapy of these aggressive cancer entities. The results of this study provide insight into real-world data on the expression of CLDN18.2 in esophageal and gastric carcinomas.

One focus of our study was to determine the clinical relevance of the tumor heterogeneity of CLDN18.2 by comparing the CLDN18.2 status of primary tumors with their corresponding lymph node metastases using different methods. Since in clinical reality only endoscopic biopsies are often available for CLDN18.2 determination in primary inoperable or metastatic carcinomas, we answered the question of whether endoscopic biopsies can provide a realistic picture of the actual CLDN18.2 expression level and how many tumor-bearing biopsies are required for this.

Our work is based on the Spotlight study, which provided one of the key prerequisites for the clinical approval of zolbetuximab. We used the immunohistochemical study antibody (Ventana, clone 43–14 A) and, in accordance with the study specifications, uniformly evaluated a carcinoma as positive if at least 75% of the tumor cells showed membranous expression of CLDN18.2 with intensity score 2 or score 3^12^.

The results of several earlier studies on the expression level of CLDN18.2 are already available, primarily in the gastric cancer. However, these studies are difficult or even impossible to compare with each other, since they used various different immunohistochemical antibodies to assess the CLDN18.2 expression; Additionally, different criteria were applied for the categorization into CLDN18.2-positive or -negative tumors (for example ≥ 40% CLDN18.2 positive tumor cells, or, in other studies, any expression levels)^13–16^.

As operable patients were also included in the Spotlight study, we examined the effect of neoadjuvant therapy on CLDN18.2 expression for the first time. Neither the neoadjuvant radio-chemotherapy according to CROSS nor the perioperative FLOT treatment had an effect on the proportion of CLDN18.2 positivity. Based on the results of the Spotlight Study and the Glow Study, approximately 33–38% of gastric carcinomas and transitional cancers are positive for CLDN18.2^8,14^. At around 28%, we found slightly fewer CLDN18.2 positive carcinomas. Thus, under “real world” conditions, it can be assumed that approximately one third of all esophageal and gastric adenocarcinomas are CLDN18.2-positive.

The heterogeneous distribution of therapeutically relevant target proteins within a tumor has already been described for Her2/neu or PD-L1^15,16^. Until now, however, reliable data on the heterogeneity of CLDN18.2 expression in esophageal and gastric adenocarcinomas did not exist. The heterogeneity is highly relevant not only for the correct classification into positive- or negative tumors, but also for the therapeutic efficacy of a drug (such as trastuzumab): The more homogeneously the therapeutic target protein is expressed in the tumor tissue, the more effective the drug will be. For example, Haffner et al. demonstrated that trastuzumab had a particularly good therapeutic effect in Her2/neu-positive gastric adenocarcinomas with a Her2/neu expression in at least 40% of the tumor cells or an (F)ISH-amplification ratio of 3 or more^17^.

For similar reasons, the GATSBY study, which tested the efficacy of a trastuzumab-based antibody drug conjugate (ADC) in gastric cancer, will have been unsuccessful^18^.

However, heterogeneously distributed clonal tumor cells also influence the diagnostic algorithm for determining the target protein on the tumor tissue: Previous studies on Her2/neu or PD-L1 demonstrated how many tumor-bearing biopsies are necessary to obtain a realistic picture of the Her2/neu- or PD-L1 status of the whole tumor: We could demonstrate that for a correct Her2/neu- and PD-L1 assessment of gastric adenocarcinomas a minimum of 5 (Her2/neu) and 4 (PD-L1) tumor-bearing biopsies are necessary^12^. It is clinically highly relevant whether endoscopic biopsies can be representative of an overall tumor to keep the proportion of false-negative classified carcinomas as low as possible.

This study is the first to address this issue for the tight junction protein CLDN18.2 in these adenocarcinomas of the upper GI tract. Since zolbetuximab will only be used in Her2/neu negative tumors, we also applied our virtual biopsy analyses to a Her2/neu-negative tumor collective.

To obtain virtual biopsies from digitized tumor samples stained for CLDN18.2, we developed an algorithm that enabled us to obtain up to 8–10 biopsies from the superficial parts of the tumor that could also be reached endoscopically. The virtual biopsy size corresponded to that of an endoscopic biopsy. This allowed us to get an impression of the heterogeneity of the CLDN18.2 expression and its potential implications for its use as a biomarker. We could answer the questions: (a) are endoscopic biopsies able to provide a realistic picture of the actual CLDN18.2 status of the whole tumor and (b) if the first question can be answered with “yes”, how many tumor-bearing biopsies are necessary? We observed a 76% sensitivity for 6 or more tumor-bearing biopsies. Fewer tumor-bearing biopsies worsened the correct detection rate of CLDN18.2 positive tumors. As for Her2/neu or PD-L1, we can therefore state for CLDN18.2 as well that multiple tumor-bearing biopsies are necessary to obtain realistic results on the biomarker status. This information is relevant for endoscopists, oncologists and pathologists. It must be clear that a classification of a carcinoma is always based on sufficiently representative tumor material.

We did not examine real endoscopic tumor biopsies but imitated them digitally. We chose this method to mimic the German guidelines, which recommend taking at least 8–10 endoscopic primary biopsies from a tumor. This procedure seemed more suitable than using actual biopsy material, which would only have been available to us in a smaller proportion of patients.

We examined the concordance between the CLDN18.2 expression status between the primary tumor and corresponding regional lymph node metastases. A discordant result was found in 18.5% of the tumors examined. Overall, the lymph node metastases are less frequently CLDN18.2 positive than the corresponding primary tumor, but there are also reverse cases. It might be assumed that the CLDN18.2 positive tumor clones are not always responsible for corresponding lymph node metastases or that they lose their CLDN18.2 positivity for unknown reasons. This is an important indication that the CLDN18.2 expression status of the primary tumor or the metastasis alone should not be used as the sole basis for making a treatment decision against zolbetuximab. In aberrant cases with a discordance, re-biopsy and re-assessment of CLDN18.2 expression, including both tumor and accessible metastasis, should be considered.

There are limitations applying to this study: This is a retrospective study of one single large tumor center. All patients included had operable esophageal or gastric adenocarcinomas. We did not analyze any tumors that had already metastasized hematogenously or peritoneally at the time of diagnosis to the extent that surgery no longer seemed possible or reasonable.

Since zolbetuximab can be used for Her2/neu negative tumors and PD-1 inhibitors are also a therapeutic option for the same indication, the question of the co-expression of claudin 18.2 and PD-L1 arises. We have determined this co-expression on whole tumor blocks. Our tumor collective shows that 45% of the tumors do not express significant PD-L1 in the tumor (CPS < 1) - this is higher than in the clinical studies as described in Checkmate 649 or Keynote 859 (18% and 22%, respectively). This is probably also explained by the fact that we investigated tumors of primarily operable patients and the tumors considered were pre-selected Her2/neu negative. Interestingly, 27% of the CLDN18.2 positive tumors show a significant expression of PD-L1 of CPS > 5 (see also supplement Fig. 2). In these patients, oncologists must decide with their patients whether they want to use zolbetuximab or approved PD1 inhibitors.

## Conclusion

This study examines the so far largest collective of adenocarcinomas of the upper gastrointestinal tract in a predominantly Caucasian patient collective (*n* = 1,283). We applied the immunohistochemical study antibody of the Spotlight study (Ventana) and its criteria for CLDN18.2 positivity (≥ 75% Score 2 or Score 3). We demonstrated a clinically relevant heterogeneity of CLDN18.2 expression within the tumor. A minimum of 6 endoscopic biopsies was necessary to minimize the probability of false negative results. 18% of the examined tumors showed a discordance between the CLDN18.2 expression in the primary tumor and the matched regional lymph node metastasis. To address this heterogeneity, the CLDN18.2 status of a tumor should therefore be determined or re-assessed in the primary tissue and, if available and accessible, in lymph node metastasis, to ensure a correct classification and optimal consecutive patient care.

## Electronic supplementary material

Below is the link to the electronic supplementary material.

Supplementary Material 1

Supplementary Material 2

## Data Availability

The data that support the findings of this study are available from the corresponding author AQ upon reasonable request.
