# Supplementary material for: The expression of the tight junction protein and therapeutical target Claudin 18.2 is heterogeneously distributed within esophageal and gastric adenocarcinoma
Source: Sci Rep. 2025 Aug 7;15:28958. doi: 10.1038/s41598-025-12337-4 (PMC12332122; doi:10.1038/s41598-025-12337-4)
Supplement: Supplementary file 1 — Supplementary Material 1 [file 41598_2025_12337_MOESM1_ESM.docx]

Supplement Figure 2: Ratio of CLDN18.2 and PD-L1 CPS status. The distribution of PD-L1 expression in four groups (CPS <1, CPS 1-4, CPS 5-10, CPS >10) in claudin 18.2 positive and claudin 18.2 negative carcinomas is shown. This comparison is based on the whole tumor blocks (n=66 in which CLDN18.2 and PD-L1 were present).
