# Supplementary material for: The expression of the tight junction protein and therapeutical target Claudin 18.2 is heterogeneously distributed within esophageal and gastric adenocarcinoma
Source: Sci Rep. 2025 Aug 7;15:28958. doi: 10.1038/s41598-025-12337-4 (PMC12332122; doi:10.1038/s41598-025-12337-4)
Supplement: Supplementary file 2 — Supplementary Material 2 [file 41598_2025_12337_MOESM2_ESM.docx]

| 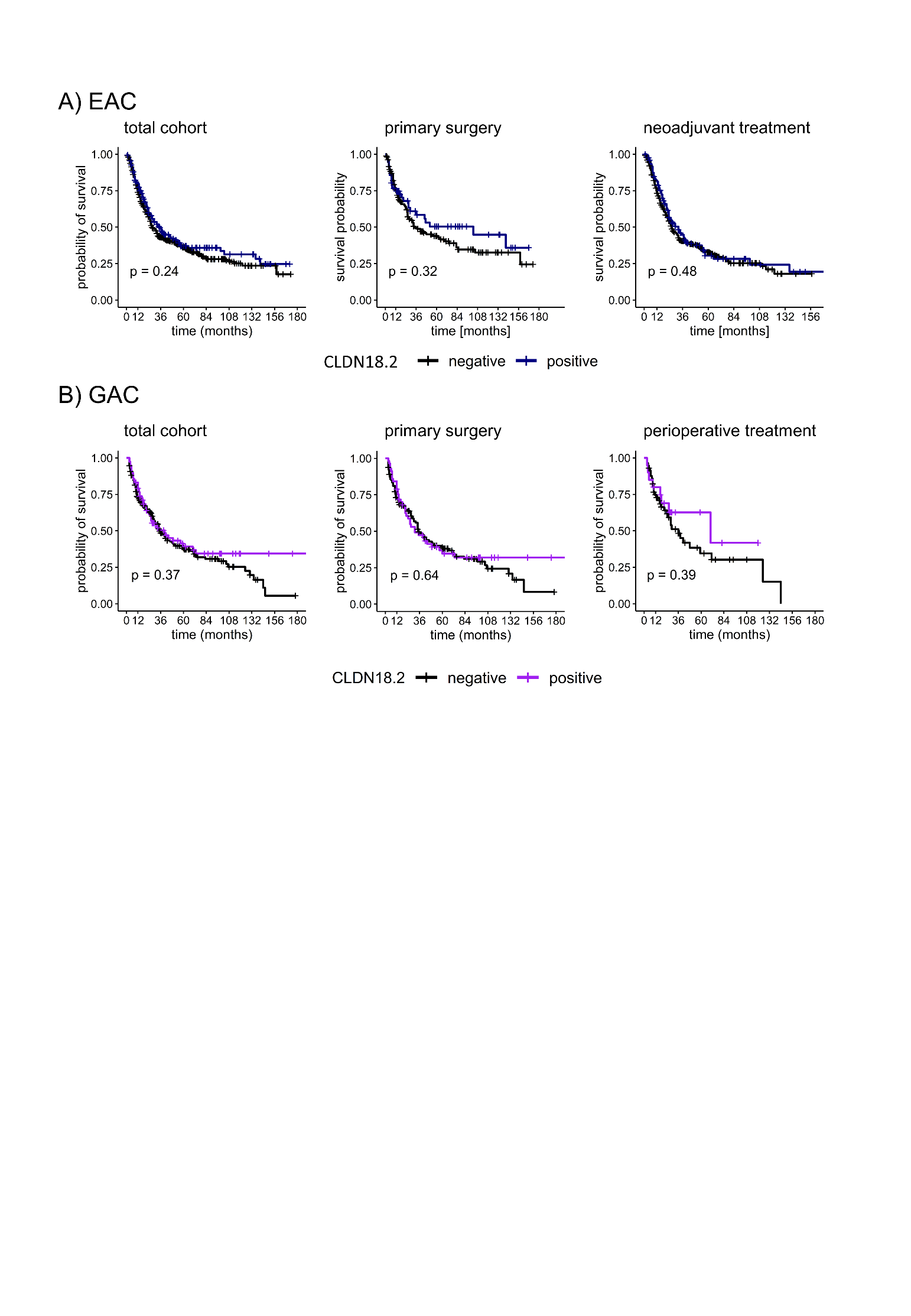 |
| --- |
| **Supplementary Figure 1: CLDN18.2 expression and overall survival in EAC and GAC cohorts**  The expression of CLDN18.2 was not associated with the overall survival in the EAC cohorts, neither in the total cohorts nor in the cohorts receiving primary surgery or the cohort receiving neoadjuvant or perioperative treatment (n= 522). |
